# A General System of Toxicology, or a Treatise on Poisons, Drawn from the Mineral, Vegetable, and Animal Kingdoms, Considered as to Their Relations with Physiology, Pathology, and Medical Jurisprudence

**Published:** 1816-05

**Authors:** 


*Traité des Poisons tirés des Regnes Mineral, Végétal, et Animal, ou Toxicologie Générale, considérée sous les Rapports de la Physiologie, de la Pathologie, et de la Médecine légale.* Par M. P. Orfila, Docteur en Médecine de la Faculté de Paris; Professeur de Chimie et de Physique. Tome Premier. 1re Partie, pp. 304. 2e Partie, pp. 326. Octavo. A. Paris, 1814.

---

*A general System of Toxicology, or a Treatise on Poisons, drawn from the Mineral, Vegetable, and Animal Kingdoms, considered as to their Relations with Physiology, Pathology, and Medical Jurisprudence.* By M. P. Orfila, M.D. &c. Translated from the French. Vol. 1, Part I. pp. 246. Part II. pp. 268. Octavo. London, 1815—1816.

The utility of a complete system of doctrine respecting poisons must be self-evident to all who have thought, or are capable of thinking, on the subject. Indeed, there are few individuals among the two great classes of which the medical republic consists, who have not had occasion, one time or other, to deplore the want of such a publication :—they, by whom medicine is practised as a money-getting art, for the sake of their own interest and character; the few, who cultivate it as a dignified science, from feelings more pure and elevated. Of all the branches of medical education, none should be more deeply studied, more philosophically understood, than this. Its influence upon the institutions of man, whether regarded in a physical or moral point of view, is equally momentous. Yet there is no practical subject upon which the notions of professional men are generally so superficial and incorrect. This truth, unfortunately, requires little illustration; or we might cite the acrimonious controversies sometimes sustained at our public tribunals, and the disgraceful scenes of ignorance and incapacity too often exhibited there. It were much to be wished that, in all our schools of medicine, toxicology should form an essential, if not distinct, branch of public instruction. Directed by such considerations, our analysis of the work of M. Orfila will be unusually extended and minute. The execution of this first volume bespeaks him eminently qualified for the task which he has undertaken—possessed of uncommon industry and precision, and all the varied acquirements as natural philosopher, chemist, and physician, necessary to reflect light upon such an investigation.

M. Orfila, in his Preface, after commenting on the difficulty and importance of the subject, states, that the whole work will consist of two octavo volumes, divided into two sections, and preceded by an introduction. In the first section will be delivered the history of the different poisonous substances belonging to the three kingdoms of nature; and their various relations to chemistry, physiology, pathology, and forensic medicine, considered. This history will be comprised under the six following paragraphs:

Exposition of the chemical properties and external characters of poisons; their physiological action. General symptoms. Organic lesions produced by them. Application of the facts developed in the preceding paragraphs to various cases of forensic medicine; and, finally, Treatment.

The second section will comprehend, in two chapters, all that relates to poisoning generally considered.—First chapter, the means necessary to prove that poison has been taken by a person yet living. Article 1. Diagnosis of the symptoms of acute poisoning from those of other diseases, and their variation, according as vomiting has, or has not, taken place, and the degree of confidence to be attached to those experiments wherein animals are made to swallow the matter ejected by persons supposed to have been poisoned. Article 2. Investigation of the class to which the poison belongs; its nature; method of chemical analysis, and order of employment of the re-agents, when the poison is in small quantity. Article 3. History of slow poisoning.—Second chapter, every thing relative to the examination of the body of an individual destroyed by poison. Article 1. Manner of proceeding to the inspection, &c. Article 2. Mode of distinguishing sudden death, determined by an internal cause, from that consequent on the action of poison. The whole will be concluded with directions for the preparation of the chemical tests mentioned in the history of particular poisons.

The Report made upon this first part of the work to the French Institute, we shall not transcribe. It has already appeared in some of the English Journals.

Having noticed the Greek origin of the term Toxicology,* M. Orfila gives the following definition of poison:

"We denominate every substance a poison, which, taken internally in a very small dose, or otherwise applied to the living body, destroys health, or utterly extinguishes life."

He then proceeds to arrange the various poisons under six classes: the corrosive; astringent; acrid; narcotic; narcotico-acrid; and putrifying or septic. The several species of the corrosive class are preparations of mercury, arsenic, antimony, copper, tin, zinc, silver, gold, bismuth, concentrated acids, pure and carbonated alkalis, caustic alkaline earths, muriate and carbonate of barytes, glass and enamel, in powder; cantharides.—The four first species occupy the *first part* of M. Orfila's work; and these only we have at present to consider.

*Section* 1. *Of Poisons in particular; their chemical properties; physiological action, and consequent symptoms; of the organic lesions they produce; and treatment.*

*Chapter I. Class* 1. *Of corrosive poisons.*—So called from irritating, inflaming, and corroding the textures with which they come in contact. Their operation varies much, according as they are applied internally or externally, in a solid or liquid form. In general, the most terrible of poisons.

*General action of corrosive poisons.*—Employed internally in minute doses, they act variously on the different organs and functions, and may be advantageously used in the treatment of diseases: largely administered, their effects are horrible; for the most part, deadly. Sometimes they are absorbed, and exert their influence on the brain, heart, and other organs: sometimes, the texture of the stomach is destroyed, and the other organs cease to act from sympathy, independently on absorption. Death, in some rare cases, results from gastric inflammation induced by the poisonous substance.

*General symptoms resulting from corrosive poisons.*—These depend almost universally on lesions of the alimentary tube, of the nervous or circulating systems. The principal are, burning and constriction of the mouth, tongue, gullet, stomach, and bowels; dreadful pains throughout the whole intestinal canal, especially the stomach and gullet; hiccough; nausea; hard vomiting, sometimes bloody, and menacing suffocation; bloody stools, with or without te-

---

* τοξικὸν, poison; and λογος, a discourse.

nesmus; pulse small, wiry, frequent, often imperceptible; icy coldness; sometimes intense heat; insatiable thirst; dysury, strangury, ischury; cold sweat; purple spots over the whole body; often a miliary eruption; sudden decomposition of the features; loss of vision; *risus sardonicus*; horrible convulsions; depravation of intellect.

*Organic lesions produced by corrosive poisons.* Inflammation, constriction, gangrene, sphacelus, and perforation of the intestinal canal, form the principal character of these lesions. Detachment of the gastric villous, from the muscular, coat is also regarded as an infallible proof of their action, which is often extended to the other viscera; and the skin covered with gangrenous spots. Sometimes, the body displays no alteration.

*General treatment of poisoning by corrosive substances.*—Three modes of obviating the destructive influence of these poisons have been adopted: Decomposition by chemical substances; the evacuating plan, followed by sedatives, antiphlogistics, antispasmodics; and, lastly, a combination of the two preceding methods. To the first of these, many objections apply: the antidotes employed are frequently inert in the stomach; or, if decomposition be effected, the new compound may still possess deleterious properties. The evacuating and antiphlogistic plan is destitute of danger; and, on all accounts, preferable.

*Article* 1. *Species* 1. *Mercurial poisons.* Of these, the six following varieties are enumerated:—1. *Corrosive sublimate* (muriate).* 2. *Red oxyde of mercury* (red precipitate). 3. *Turbith mineral* (sub-sulphate). 4. *The various nitrates of mercury.* 5. *All the other mercurial preparations,* mercurius dulcis (submuriate) excepted. 6. *Mercurial vapours, or the metal in a state of extreme division.*

Of all the preparations of mercury, the muriate is that with the chemical characters and operation of which, it behoves the juridical physician to be most intimately acquainted. From its common employment, in the hands of the ignorant and careless, fatal consequences are continually resulting. It often forms the deadly instrument of the suicide and the murderer. We shall, therefore, pass over all the other less important varieties just specified, in order to concentrate our whole attention upon this tremendous poison, and develope its chemical properties, and in-

---

* *Oxymuriate of mercury,* of the London Pharmac.; *Ducto-muriate* of French chemists; and *deuchloride* of the British.—Rev.

fluence on the animal economy, with a minuteness which our limits would otherwise have forbidden.

*Chemical history of corrosive sublimate.* An acid metallic salt, formed of muriatic acid and oxyde of mercuryat a maximum of oxydation. Its physical characters must be sufficiently familiar to our readers. Taste acrid, caustic, highly styptic, producing a sense of constriction of the throat. Specific gravity 5,1398. Volatilized, when thrown upon burning coals, and exhaling a dense white smoke, of pungent, not alliaceous odour, which irritates the throat and nose, and often excites cough. A clean plate of copper is tarnished by it; and assumes, on friction, a white and shining hue. It reddens litmus paper in consequence of the excess of acid. Soluble in eleven parts of cold, and in two of boiling water. This solution is transparent, colourless, and inodorous; of a styptic metallic taste; turning paper and infusion of litmus red, syrup of violets green; precipitated of a yellowish red by pure potash in alcohol;* of a very deep brick red by carbonate of potash; of a clear brick colour by the sub-carbonate; deepish yellow by lime-water in small quantity (changing to red if the quantity of alkali be increased, and again to beautiful yellow on a farther addition); white by ammonia, by muriate of tin, and prussiate of potash (the white colour of this last precipitate successively changes, in a few hours, to yellow and clear Prussian blue); black by sulphuret of ammonia, sulphuretted hydrogen, and hydro sulphuretted water. Nitrate of silver yields to it a white, heavy, insoluble precipitate (muriate of silver). The supernatant fluid contains nitrate of mercury. A plate of copper, or of zinc,† by stronger affinity for muriatic acid, decomposes the solution, when immersed in it. Metallic mercury precipitates the sublimate, and converts it into submuriate. All vegetable substances act upon the solution by disengaging the muriatic acid,‡ and precipitating the salt, in form of submuriate, combined with vegetable matter. All these precipitates, heated with potash, yield metallic mercury.

* This colour will vary according to the quantities of potash and sublimate employed. If the solution of the latter be weak, for instance, at 1 deg. of Baumé's areometer, (erroneously marked 10 deg. by the Translator) the precipitate will be white, brick, or rose-coloured.—Rev.

† The fluid in which the zinc plate is immersed, will contain, instead of the sublimate, *muriate* of zinc, not *sulphate* of zinc, as stated by the Translator.—Rev.

‡ The Translator has falsely rendered it muriatic *gas*.

*Burgundy wine*, mixed with a solution of sublimate in distilled water, remains clear. Potash throws down from the mixture a black precipitate; ammonia, a very dark green; prussiate of potash, a white changing to violet. Syrup of violets is reddened by it. The plate of copper, and hydro-sulphurets, act upon it as on the simple solution. Hence, if the stomach of a person destroyed by poison contain wine, no attention should be paid to the action of those tests which change the colour of the precipitates. Upon adding more sublimate to the wine, it becomes turbid, and yields a violet precipitate.

*Albumen* powerfully decomposes sublimate, forming with it a white, flaky precipitate, soluble in excess of albumen, and yielding to the action of heat, in a glass tube, a light incinerated substance and metallic mercury. To this interesting fact we shall presently recur. — On mixture with *gelatine*, the solution of sublimate becomes turbid; and a white, solid, gelatinous matter is precipitated. This disappears, and the mixture becomes transparent, on raising its temperature to the boiling point. Hence gelatine effects the same decomposition of sublimate as albumen, changing it into submuriate, which combines with a portion of animal matter.—*Ozmazome*,* dissolved in water, affords with sublimate a reddish yellow precipitate, becoming red on desiccation.—Solutions of *sugar of milk*, of *resin of bile*, and of *picromel*,† remain clear on mixture with sublimate; but the picromel yields, after some days, a scanty whitish precipitate. —*Fibrine* decomposes sublimate, throwing down from an aqueous solution the submuriate. The presence of muriatic acid may be detected in the remaining fluid.

*Milk* shews no visible change on mixture with concentrated solution of sublimate. If one part of the solution be added to fourteen of milk, the mixture assumes a sky-blue colour with syrup of violets; blackish grey with caustic potash; yellow, afterwards changing to blue, with prussiate of potash;‡ and black with hydro-sulphuret of

---

* An animal substance, which forms a constituent of muscle, and gives the peculiar smell and flavour to broth. Hence its name.—Rev.

† A constituent of the bile of some animals; by which the resin of bile is rendered soluble in water.—Rev.

‡ By inaccuracy in punctuation, the translator has made an egregious mess of this passage. According to him, the milk turns blue on being added to the sublimate; syrup of violets changes the mixture to a blackish grey; caustic potash, to a yellow; and prussiate of potash, to a blue!—Rev.

ammonia. The plate of copper operates as on the simple solution. If the quantity of solution employed be seven or eight times greater than that of the milk, a white coagulum is formed; which, when dried, becomes solid, yellowish, and unalterable by the action of air or water. It consists of submuriate and the caseous and butyraceous parts of milk, and yields metallic mercury on being heated.

*Common broth,* strained and limpid, grows lightly turbid on mixture with a small dose of sublimate in solution; but yields no precipitate. Caustic potash precipitates it of a white, grey, or black colour,* instead of yellow, as would happen if there were an excess of sublimate; lime-water, a dead or yellowish white. The plate of copper, syrup of violets, prussiate of potash, ammonia, nitrate of silver, and hydro-sulphurets, produce the same changes as on the aqueous solution. But, if five or six parts of sublimate be mixed with one of broth, a white, heavy, flaky precipitate immediately results, in which submuriate is contained, and which yields, on exposure to heat in a glass tube, metallic mercury and animal matter. Hence broth possesses, like all animal substances, the property of converting sublimate into submuriate of mercury.

*Human bile,* diluted with an equal quantity of water, and mixed with a tenth part of concentrated solution of sublimate, yields a copious reddish yellow precipitate. The same thing happens, but more slowly, when the bile is largely diluted. This precipitate is in the form of a reddish powder, composed of submuriate and animal matter; and yields metallic mercury on the application of heat. Sometimes, however, no precipitation takes place. This depends upon the different and ever-varying principles of which bile is composed. But if the mixture be allowed to stand for a time, it will at length become turbid, and deposit submuriate in combination with animal matter. From a mixture composed of one grain of sublimate, and one drachm of bile, dissolved in one ounce of water, ammonia and potash produced no precipitate; while these alkalis precipitated a solution of the same strength without bile. But the sulphuretted alkali and lime-water precipitated a solution which the (other) alkalis did not render turbid.

*Action of corrosive sublimate taken internally.* How does this poison, administered in large doses, operate? and

* Erroneously *red* by the translator.—Rev.

upon what organ is its deadly influence first exerted? Such are the queries proposed by M. Orfila, after tracing the effects of sublimate when introduced into the system in a dose not directly fatal. From the experiments of Brodie, here quoted, it appears that sublimate exerts a corrosive action on the stomach, and that death ensues from propagation of this action to the heart and brain. Inflammation of the stomach could not so soon destroy life; and absorption must be prevented by the state of its mucous membrane. The observations of Lavort concur to do away the idea that death results from introduction of the poison into the absorbent system.

*Symptoms peculiar to poisoning by corrosive sublimate.* In illustration of these, seven cases, wherein the poison was internally or externally administered, are detailed; and from them is deduced the following assemblage of phænomena which characterize its terrible operation upon the human organs:—Acrid, styptic, metallic taste; sense of constriction and burning heat in the throat; anxiety; rending pains in the stomach and whole intestinal canal; nausea; frequent and hard vomitings of a fluid, sometimes bloody; diarrhœa; sometimes dysentery; pulse small, wiry, frequent; lipothymia; general debility; dyspnœa; cold sweat; spasms of the limbs; insensibility; convulsions; death.

*Lesions attributed especially to corrosive sublimate.* It has been asserted by some that this poison never produces perforations of the alimentary tube; exerts no action on the mouth or œsophagus; that it destroys, burns, and detaches the gastric mucous membrane, without changing the muscular; extends its ravages to the cæcum, and excites no cutaneous eruption. By M. Orfila, these positions are deemed inadmissible. He declares it incontestibly proved, that general inflammation and perforation of the intestinal tube may be produced by all the corrosive poisons; the mucous membrane of the stomach be detached by many of them; and gangrenous patches of the integuments result from all those of more active operation. In the present state of our science, therefore, the seat, extent, and character of the lesions produced by corrosive sublimate, cannot be precisely indicated.

*Application of the preceding facts to different cases of poisoning by corrosive sublimate.* There are four different combinations of circumstances under which, in such cases, the medical practitioner may be called upon to act. Rules of conduct, applicable to each, are delivered by M. Orfila.

We shall trace them as briefly as their high value, and the importance of the subject, will admit.

1. *The patient is living: the remainder of the poison may be procured.* Here the condition of the patient, the information which he and his attendants may afford, and chemical examination of the suspected poison, are chiefly to be relied on. If the poison be liquid and in small quantity, a little should be dropped from a quill, or small glass tube, on litmus paper and a clean copper-plate. It should afterwards be successively tried with concentrated solutions of hydro-sulphuret of ammonia, nitrate of silver, pure and carbonated potash, ammonia, prussiate of potash, and syrup of violets. A portion of it, if any be left, may also be mixed with pure potash, evaporated to dryness in a small porcelaine capsule, and the residue raised gradually to red heat in a glass tube; whereby globules of metallic mercury will be obtained. Lastly, a plate of zinc, immersed in the solution, will decompose it. If the liquid be mixed with milk, broth, tea, wine, or syrups, the mixture may be slightly turbid without distinct precipitate, or perfectly clear and yield a precipitate. This will depend on the quantity of sublimate. Now, either the liquid will present the usual phænomena with the tests just indicated, or the precipitates furnished will be modified by mixture with the different substances. If there be a precipitate, metallic mercury may be obtained by calcination of the desiccated mass in a small glass tube. If the salt be in a solid form, metallic mercury will appear on heating it with potash or metallic antimony; and an aqueous solution of it will answer to the ordinary chemical tests. If, lastly, the poison form part of a plaster, the latter should be cut into small pieces, boiled, for fifteen minutes, in distilled water, and the filtered liquid subjected to the usual tests. No sublimate being detected here, the whole solid portion should be dried in a capsule, mixed with potash, and introduced into a glass retort with a long-necked receiver, and gradually heated to redness. Thus metallic mercury will be evolved, adhering to the neck of the retort, and mixed with thick blackish oil. But the quantity may be so minute as to elude observation. In such case, the neck of the retort must be broken into small pieces, and washed with perfectly pure nitric acid. The metal, thus reduced to the state of nitrate at minimum, is readily recognized by the red, white, and black precipitates, which it forms with chromate of potash, muriatic acid, ammonia, and the hydro-sulphurets. Thus, the examiner may announce decidedly the presence of a mercurial poison: but, in order to prove incontestibly that the poison is sublimate, it is necessary that muriatic acid be obtained; and this may be done by submitting the plaster to the action of caloric in a proper apparatus.

2. *The patient living: all the poison swallowed: the matter vomited may be examined.* This case is more difficult, and more frequent of occurrence, than the former; hence it demands profound knowledge and attention. If the matter vomited be fluid, unmixed with aliment, and, when filtered, answer to the ordinary tests, no doubt of the presence of sublimate can exist: but if the precipitates vary, or do not all shew themselves, it should be treated with caustic potash, dried, and heated to redness in a small glass retort, to which a spherical receiver has been adapted.* The appearance of mercurial globules will announce the nature of the poison: but if the globules do not appear, and the neck of the retort, treated with nitric acid, furnish a liquid in which nitrate of mercury at minimum is detected, the inference will be equally obvious. This is the only good method of analyzing a vomited fluid. — If the matters ejected be partly liquid, partly solid, they should be separated by straining; and the solid portion, preserved in alcohol, be afterwards dried and calcined, in order to obtain metallic mercury; the liquid having been previously examined by the usual tests. Albumen, milk, broth, and other alimentary substances, have the property of readily converting sublimate into muriate at a minimum (submuriate): hence we frequently cannot detect its presence in the fluid ejected by vomiting. In this case, the affusion of lime-water on the remaining solid matter has been proposed; in the expectation that, if the submuriate were combined with it, a black colour would be produced. M. Orfila rejects this experiment as inconclusive, for the solid matter vomited may itself be black; and it is proved by direct experiment, that affusion of lime-water on alimentary substances containing the submuriate, does not invariably produce a black colour. And, even when the alimentary mass becomes black from the action of lime-water, we

* It has pleased the Translator, somewhat whimsically, to attach a *balloon* to his retort, instead of a *receiver*. Persons, unacquainted with the phraseology of French chemistry, would imagine that he was meditating an aërial excursion with his retort, and going to pursue his analytical researches among the clouds. Rev.

cannot positively infer that it results from the decomposition of the submuriate; unless that mass yield to cold nitric acid the black oxyde colouring it, and thus form a colourless nitrate at minimum, precipitated black by the hydro-sulphurets and ammonia; red by chromate of potash; and white by muriatic acid.

3. *The patient living: whole of the poison swallowed: the matter vomited cannot be examined.* Here chemistry affords no light. The juridical physician must be guided in his diagnosis by the condition, circumstances, and habits of his patient; nature of the attack; season of the year; and character of the prevailing diseases.

4. *The patient dead.* Supposing that neither a remnant of the poison, nor the matter vomited, can be examined, dissection and chemical analysis of the parietes and contents of the alimentary tube can only be had recourse to. In this examination, ligatures should be passed round the œsophagus, rectum, and biliary ducts; the intestinal canal removed, and slit up the whole length; all its contents preserved; the internal surface washed in distilled water; the inflamed, sloughy, gangrenous portions, all the parts surrounding any perforation, cut out, and preserved in alcohol. The usual methods of detecting sublimate in the intestinal contents and in the distilled water failing, the preserved portions of alimentary canal should be mixed with potash, and calcined in a retort, to obtain metallic mercury. M. Orfila next proves by experiment, the possibility of detecting the poison in combination with the texture of the animal organs. The intestinal canal operates, like all other animal substances, by disengaging muriatic acid from the sublimate, and converting it into submuriate, which readily enters into combination with such texture.

*Treatment of poisoning by corrosive sublimate.* After stating the question, "Exists there any known antidote of corrosive sublimate?" M. Orfila proceeds to demonstrate, by conclusive experiments, the inefficacy of the substances extolled as such by Navier; alkaline salts and earths; sulphurets of potash and lime; and alkaline chalybeate tinctures. He proves that the yellow oxyde, resulting from decomposition of sublimate by potash, soda, and lime, acts upon animals as a virulent poison; and that the black sulphuret, produced by the action of the sulphuretted alkalis, is almost equally destructive in its operation. Sulphuretted hydrogen gas, hydro-sulphuretted water, sugar, infusion of cinchona, metallic mercury, broth, and albumen, are next examined in succession. The five first are shewn not to act as antidotes of sublimate. Broth decomposes it, but not with sufficient energy; and merely retards its deleterious operation. Albumen is the real antidote; it combines with the sublimate, forming a triple compound of albumen, muriatic acid, and oxyde of mercury at minimum; which is innocent even in very large doses. This most important fact is illustrated by numerous experiments; and M. Orfila concludes by observing that, of all the substances hitherto proposed as antidotes of corrosive sublimate, albumen, largely administered, is alone useful; as it may be taken with impunity, forms with the poison an inert compound, and may be always readily procured. From the first appearance of the symptoms, the patient should, therefore, swallow several glasses of white of egg (albumen) diluted with water. Decoction of linseed, marsh-mallow root, or of mallow leaves; rice-water, sugared water, gelatinous broths, or even simple water, at the temperature of from 25° to 30°* may be given, if eggs cannot be obtained. These should be copiously administered until vomiting is induced, and continued as long as that operation takes place. If from peculiarity of constitution, or the existence of *trismus,* the patient cannot vomit, his stomach may be emptied by an elastic gum tube, introduced thro' the mouth or nostril, and fitted with a syringe. The efficacy of all these fluids, it must not be forgotten, depends upon their bulk. They should be administered even when the patient feels no inclination to vomit; and are greatly preferable to emetics for evacuating the stomach; as the irritation, already excited, is soothed, rather than aggravated by them. Oils and fat substances should never be employed. If abdominal inflammation exist in its first stage, general and local bleedings; emollient and anodyne glysters; hot fomentations, should the soreness of the abdomen not render them insupportable; immersion of the lower half, or whole, of the body, for some hours,† in the warm bath, with care to keep up its original temperature; and low diet, are indicated. If the inflammation be far advanced, gangrene may be dreaded:

---

* Of the centigrade thermometer, commonly employed by Orfila; and of course, equivalent to 77—86 of Fahrenheit's scale. This the Translator should have explained.

† Where the Translator learned that the French *demi-bain* should be rendered "*pediluvium,*" we are at a loss to imagine. Rev.

here bleeding is inadmissible. The treatment should be that of common intestinal inflammations. Antispasmodics and narcotics may be prescribed, if there arise spasms or convulsions. The patient, during convalescence, is to be supported with milk, farinaceous substances, jellies, and light animal broths. If he have been suffering from illness at the time, regard should be paid, in the treatment, to the character and complication of the pre-existing affections.

*Article* 2. *Species* 2. *Arsenical Poisons.* The following eight varieties are successively examined: 1. *Arsenious acid* (white oxyde); 2. *Arsenites* (combinations of that acid with salifiable bases); 3. *Arsenic acid*; 4. *Arseniates* (combinations of arsenic acid with various bases); 5. *Yellow sulphuret of arsenic*; 6. *Red sulphuret*; 7. *Black oxyde*; 8. *Arsenical vapours.*

From the motives specified in our last article, when speaking of corrosive sublimate, we shall restrict our observations principally to the arsenious acid; after slightly mentioning some circumstances, with respect to the pure metal, perhaps not correctly understood by some of our Readers. *Metallic arsenic* is solid, of a steel-grey color, shining when recently prepared; texture grained, sometimes scaly; hardness inconsiderable; fragility great; specific gravity 8,308. Heated in a close vessel, it sublimes and chrystallizes without fusion or alteration. It combines readily with oxygen. Given to dogs, in the dose of a drachm, it has frequently produced no sensible effect; and hence been considered as not poisonous: but, in other instances, its operation has proved destructive. This "probably depends on the facility with which it is converted into an oxyde."

*Chemical history of arsenious acid.* — Commonly found in white masses; opaque externally; yellow, transparent, and vitrified internally; taste acrid and corrosive; specific gravity 5,000. Thrown on burning coals, it exhales dense white vapours, of an alliaceous smell, which cover a plate of copper held over them with a layer of beautiful white, readily detached by the finger. The acid is soluble in water. One thousand parts of the fluid, at the temperature of 12 deg. Reau.* dissolved, in twenty-four hours, two parts and a half of the acid. Seventy-seven

---

* 12 deg. Reaumur, equal to 59 deg. of Fahrenheit's scale. Rev.

parts and a quarter are taken up by one thousand of boiling water. Lastly, if a certain portion of the acid be boiled in water, the superfluous quantity is deposited in tetrahedral prisms, on suffering the liquid to cool; and a thousand parts of the latter retain in solution thirty of the former. This solution is colourless and inodorous; scarcely affects the tinctures or papers of litmus or turmeric; turns syrup of violets green; restores the colour of litmus paper reddened by an acid; forms soluble arsenites with potash, soda, and ammonia; with lime, a beautifully white arsenite of lime; and is precipitated, by sulphuretted hydrogen gas and hydro-sulphuretted water, of a golden yellow. By these means, the acid may be detected in a solution containing only $\frac{1}{100000}$. This precipitate may be decomposed by drying and heating it with pure potash in a glass tube, whereby sulphuret of potash and metallic arsenic will be obtained. The former may be recognized by the odour exhaled on the addition of a drop of water. The hydro-sulphurets only affect the solution when a little nitric or muriatic acid is added to the mixture. In this case they afford the same gold-yellow precipitate (sulphuret of arsenic). The sulphuret of potash, dissolved and in small quantity, affords a white precipitate; or yellowish, if the dose be more considerable. The nitrate of silver is thrown down yellow, and blackens on exposure to light. Sulphate of copper in solution affords a green flaky precipitate; upon adding an atom of liquid potash to the mixture, the green precipitate is instantly produced. Ammoniaret of copper gives a green precipitate; and the acetate a green, inclining to yellow. The former of these two is the most delicate test which copper furnishes for the detection of arsenic; as by it the acid may be discovered in a solution containing but $\frac{1}{110000}$ of its weight. Red mineral cameleon (potash and oxyde of manganese melted) assumes with it a yellow colour. Prussiate of potash, albumen, gelatine, sugar of milk, picromel, and resin of bile, affect not the arsenical solution. But it is decomposed by the action of the galvanic pile; and metallic arsenic is deposited on the negative wire. Arsenious acid, powdered, dissolves wholly by ebullition in pure muriatic acid. This solution, limpid and yellow, deposits, on cooling, much arsenious acid; and after this deposition has ceased, water produces a fresh precipitate. This solution yields to prussiate of potash, when agitated, a sky-blue precipitate; when at rest, a white one, mixed with points of sky-blue and faint rose colour. Arsenious acid, pulverized and mixed with its bulk of charcoal and potash, gives out metallic arsenic upon being submitted to heat in a glass tube. This method is preferable to that wherein the arsenious acid is mixed with unctuous substances, or to the process recommended by Bostock for its decomposition.

*Decoction of Tea*, mixed with an equal part of solution of arsenious acid, produces no change. Ammoniaret of copper, without rendering it turbid, alters the mixture to a reddish violet colour. Nitrate of silver gives a yellowish white precipitate, immediately becoming black; lime water, a dirty canary yellow; sulphuretted hydrogen, a fine yellow. The latter test alone, therefore, can detect the presence of the poison in combination with tea.

*Coffee*, mixed in an equal quantity, does not disturb the solution. The mixture is precipitated of a deep yellow colour by nitrate of silver; grass green* by ammoniaret of copper; golden yellow by sulphuretted hydrogen; and yellow by lime-water. Hence ammoniaret of copper is the decisive test of arsenious acid mingled with coffee.

A mixture, formed of ten parts of *wine*, and one of the arsenious solution, is transparent, and precipitated of a deep yellow, by sulphuretted hydrogen; blackish blue by ammoniaret of copper; white by nitrate of silver. A mixture of ten parts of wine, and seven of the solution, is precipitated of a golden yellow by sulphuretted hydrogen; green by ammoniaret of copper; and white by nitrate of silver. Consequently, the latter is of no use as a test here, in either case; nor the ammoniaret of copper when the poison is largely diluted with wine.

*Solutions of albumen and gelatine* are not rendered turbid by arsenious acid. The mixture is precipitated white by nitrate of silver: and affected as the aqueous solution by other tests.

No change is observed on mixing equal parts of *broth* and arsenious solution. Ammoniaret of copper changes the colour of the compound to a dirty green without precipitation. Nitrate of silver throws down a white precipitate. Lime-water and sulphuretted hydrogen operate as on the simple solution.

*Human bile* and the arsenious solution do not grow turbid on mixture: and the liquid is precipitated by the four

---

* "*Vert de pré*," the translator, with his wonted negligence, has renered, "*nearly green*. Rev.

tests recently mentioned, as though the solution were unmixed with bile.

The white colour of a mixture of ten parts of *milk* and one of the arsenious solution is changed by sulphuretted hydrogen into canary yellow; by ammoniaret of copper, into a light green. Nitrate of silver effects no alteration. The hydro-sulphurets afford a golden yellow precipitate, provided a drop or two of nitric or muriatic acid be employed; the ammoniaret of copper a green, and nitrate of silver a white one, whatever be the quantity of arsenious acid.

The fluid contained in the stomach of a rabbit, which had been destroyed by three grains of arsenious acid in solution, was precipitated white by nitrate of silver; greyish white by lime-water; green by ammoniaret of copper, and deep yellow by hydro-sulphuretted water.

Mixtures of arsenious acid with *wine, tea, coffee, broth, albumen, gelatine, and milk,* evaporated separately, and treated with boiling distilled water, have constantly yielded a fluid in which the presence of the acid might be detected by one of the four tests presently to be named. Some of them, however, furnish a different precipitate from that which they produce with the simple arsenious solution. The sulphuretted hydrogen gives almost always a yellow precipitate; ammoniaret of copper, less constantly, a green. Those resulting from lime-water and nitrate of silver, are much more variable. All these mixtures evaporated to dryness, and calcined with potash and charcoal, yield metallic arsenic.

*Action of arsenious acid upon the animal œconomy.* The experiments of our able physiologist Brodie,* are here quoted to prove that arsenic does not, in general, destroy life by producing inflammation of the stomach; that its influence is exerted upon the nervous system, the organs of circulation, and alimentary canal; and that death immediately results from suspension of the functions of the heart and brain. Yet, in some cases, where the subject survives the immediate operation of the poison, and gastric inflammation has time to develope itself, life may, doubtless, be destroyed thereby.

---

* See "Philosophical Transactions" for 1812. M. Orfila positively declares, that he himself, considers the opinions of Mr. Brodie respecting the action of arsenic, much more correct than those generally received. Our accurate translator has penetration enough to discover, that M. Orfila does "*not*" so consider them. REV.

*Symptoms of poisoning by arsenious acid.* In illustration of these, four examples are cited from different authors. The last of them is the case observed by Roux, consequent on application of the arsenical paste, to which we alluded upon reviewing the work of Mr. Cross in our last number. The following constitute the general assemblage of phænomena observed in such cases : austere taste and fœtor of the mouth; frequent ptyalism; constant spitting; constriction of the pharynx and œsophagus; teeth set on an edge; vomiting of a matter brownish or bloody; anxiety; frequent faintings; heat in the præcordial region; inflammation of the lips, tongue, palate, throat, œsophagus; stomach so painful as not to bear the mildest liquids; stools blackish and horribly fœtid; pulse small, frequent, concentrated, irregular, some times slow and unequal; palpitations of the heart; syncope; insatiable thirst; universal heat of the body; sensation of a devouring fire; sometimes icy coldness; dyspnœa; cold sweats; urine scanty, red, and bloody; alteration of the features; livid circle around the eye lids; swelling and itching of the body covered with livid spots, and sometimes with a miliary eruption; weakness; loss of feeling, particularly of the hands and feet; delirium; convulsions, often with intolerable priapism; falling off of the hair; separation of the epidermis; and death. All these symptoms, however, are rarely seen combined. In the second history quoted by Orfila, few of them existed : and Chaussier has recorded a case wherein the only morbid sign, induced by fatal ingestion of arsenious acid in lumps, was slight syncope.

*Organic lesions attributed particularly to arsenious acid.* It is incontestibly proved that this poison may destroy life without leaving a trace of its action upon the intestinal canal. Sallin himself, who has asserted, that the morbid alterations produced by arsenic, display, like the effects of sublimate, a peculiar character, admits this fact. In general, however, the mouth, œsophagus, stomach and intestines are inflamed; the stomach and duodenum exhibit gangrenous spots, sloughs, and complete perforations; the gastric mucous membrane is destroyed and reduced to a brown reddish paste : and all the other viscera are more orless inflamed. Yet all these appearances do not prove the fact of poison having been swallowed : they serve only to corroborate the inferences drawn from chemical analysis of the intestinal contents. With respect to the state of the stomach of those poisoned by arsenic, Mr. Brodie has observed that the inflammation of this organ is sometimes extremely slight; that it, in general, directly follows ingestion of the poison, and is more intense in proportion as life is longer protracted; that it is less violent in herbivorous than in carnivorous animals; that it never extends to the œsophagus* or pharynx; and that its progress is much more vehement and rapid when the poison has been applied upon an ulcerated surface, than when taken internally. The blood-vessels of the stomach are gorged; but the inflammation is commonly confined to the mucous membrane, which is bright red, pulpy, and readily separable from the muscular; and neither sloughs nor ulceration are discovered, unless the subject of experiment have survived, for a considerable time, the reception of the poison.

*Application of the preceding facts to different cases of poisoning by arsenious acid.* The impracticability of decomposing this acid by alimentary substances at the ordinary temperature; the numerous means which chemistry affords, of distinguising it from others; and the facility of reproducing metallic arsenic, are conditions which render the solution of this problem comparatively easy.

1. *The Patient living: the remainder of the poison can be procured.* If the substance under examination be solid and pulverized, half a grain should be dissolved in half an ounce of distilled water, and the temperature raised to 80°†. This solution, tried with ammoniaret of copper, hydro-sulphuretted water, the hydro-sulphurets, nitrate of silver, red solution of mineral cameleon, lime-water, and syrup of violets, will display the changes before indicated. Another portion may be calcined with an equal volume of powdered charcoal and subcarbonate of potash; whereby metallic arsenic will be produced, yielding, when burnt, alliaceous vapours, and precipitating ammoniaret of copper, green. If the quantity of arsenic thus obtained, be very minute, forming merely a thin layer of tarnished grey powder adherent to the sides of the tube, the fragments of glass should be carefully collected; part of them submitted to the action of ammoniaret of copper, and the other placed on burning coals: thus, the characteristic phænomena will be developed. In order to acquire greater certainty, the solution of arsenious acid

* Here is variation in the opinions of M. Orfila and the British Physiologist. Rev.

† 176 deg. Fahr. Rev.

may be submitted to the action of the galvanic pile: the results of this process we have elsewhere stated. If the poison be in a lump, its physical characters should be rigorously ascertained previously to pulverization.

If the acid form part of a plaster or other external application, one portion of such medicament, properly divided, should be treated with six or seven times its weight of boiling distilled water; and the filtered solution tried by the usual tests. The other portion may then be calcined with charcoal, to obtain metallic arsenic. This result will be decisive where the operation of the other tests has proved equivocal, with the solution.

2. *The patient living: whole of the poison swallowed: the matter vomited may be examined.* If the matter be liquid, a part should be filtered, and submitted to the ordinary tests; the remainder, after adding half an ounce of potash, be evaporated to dryness, and calcined with charcoal in a glass tube. If the tests display not the peculiar characters of arsenic in the filtered liquid, it should be evaporated, and the remnant tried by calcination in the way just indicated. Again, if the matter vomited consist of both liquid and solid, the former should be treated by the usual tests; and the latter searched for the solid poison, which, if found, must be preserved for analysis. If there be none, then the mass should be divided into two portions; one of which may be treated by boiling distilled water and the common tests; the other by calcination with charcoal and potash. Should the quantity of matter be too large to admit of the operation in a glass tube, a stone retort, coated with a mixture of clay and sand, and fitted with a spherical receiver, may be employed for the process. Lastly, if the matter vomited be liquid and very abundant, and precipitated yellow by sulphuretted hydrogen, it should be mixed with an excess of hydro-sulphuret of ammonia and a little muriatic acid. Thus, all the arsenious acid will be decomposed and converted into yellow sulphuret. This sulphuret, obtained by filtration, dried and calcined with potash, will give out metallic arsenic; the potash and sulphur uniting to form a fixed compound. This process is preferable to evaporation, in operating upon a large quantity of fluid.

3. *The patient living: whole of the poison swallowed: matter vomited cannot be examined.* Close observation of the attendant symptoms; investigation of their causes; and inquiry into the habits of the patient, can only elucidate this intricate case.

4. *The patient dead.* M. Orfila, having reviewed the processes recommended by Hahnemann, Roose, Roloff, and Fischer, describes, as preferable to all others, the following "*method of discovering arsenious acid after the death of an individual poisoned by this substance.*" The intestinal canal should be detached in the manner indicated in the last article, and all the contents of the stomach collected. Any fragment of arsenic that may be found, should be removed for analysis. None appearing, the liquid should be strained off, and examined in the usual way. If no poison be detected, the solid matter should then be operated upon; all the organic lesions of the intestinal canal having previously been noted, and the stomach cut into pieces and preserved in alcohol.

The solid matter is then to be boiled for an hour in ten or twelve times its weight of distilled water, which should be supplied as fast as it evaporates. Of this liquid, when cold and decanted, a few drops should be added to the solutions of ammoniaret of copper, hydro-sulphuret of ammonia, nitrate of silver, and mineral cameleon; and lime-water. If the precipitates resulting indicate that the solution contains arsenious acid, it should be mixed with potash, evaporated, and the product calcined with charcoal. But if the liquid exhibit no trace of the poison, the solid mass should then be treated by potash and nitric acid, as recommended by Roose.* The excess of acid of the clear yellow liquid thus obtained, should be saturated with potash; and thus, if the mass really contained arsenious acid, arsenite of potash will result. The tests, just specified, will detect the most minute portions of the acid or arsenite in this fluid. If the precipitates are such as indicate the existence of this poison, it should be precipitated by hydro-sulphuret of ammonia and a few drops

* The process of Roose is thus described: The stomach should be cut in pieces and boiled, for some time, with the addition of from two to four drachms of caustic potash. Thus, the stomach will be partly dissolved, and the arsenious acid saturated with the alkali. This liquid is now to be filtered, boiled, and nitric acid gradually added, until it assumes a clear yellow colour. It is then to be filtered anew, and the excess of acid saturated by an alkaline carbonate. The carbonic acid is to be driven off by boiling, and the liquid precipitated by boiling lime-water. This precipitate contains arsenite of lime, and perhaps a little arseniate resulting from conversion of a portion of the arsenious into arsenic acid by the nitric acid. Washed, dried, and heated to redness in a retort, it yields metallic arsenic. Roose recommends the addition to the precipitate of half the quantity of boracic acid, with a view of favouring the separation and decomposition of the arsenious acid.

of nitric acid. Thus yellow sulphuret of arsenic will be obtained; from which all the metal may be recovered by drying on a filter and mixing it with its bulk of subcarbonate of potash, and melting in a glass tube. The solid matter yielding no trace of arsenious acid to this treatment, the stomach itself should next be subjected to analysis.

But a different route must be followed, if sulphurets or hydro-sulphurets have been swallowed by the patient to controul the operation of the poison: for, if the arsenious acid have been decomposed by such means, it will be converted into the yellow sulphuret, possessing characters different from those of the acid. Hence the following processes should be adopted: 1. If the stomach contain only liquids, they should be collected and allowed to deposit the yellow insoluble part. This, dried on a filter and thrown upon burning coals, will exhale a mixed smell of sulphur and garlick. Another portion should be completely pulverized with an equal bulk of subcarbonate of potash, and calcined in a glass tube to obtain metallic arsenic. 2. If solid matter be found in the stomach, mixed with particles of yellow sulphuret of arsenic, two drachms of subcarbonate of potash should be added, and the whole evaporated in a porcelain capsule. The mass resulting should be pulverized and tried by calcination. If the quantity of matter be too large to be contained in a glass tube, the process may be performed in a stone retort, fitted with a bitubulated receiver, in order that the gas, extricated from decomposition of the animal matter, may escape by one of the tubes. After all, it is sometimes impossible to detect the presence of arsenic in the stomach of persons unquestionably destroyed by this poison.

*Treament of poisoning by arsenious acid.* From the experiments of Renault, made upon dogs, it appeared that the *alkaline sulphurets* are of no use in this kind of poisoning; in fact, that "animals die as soon or sooner when the pretended antidote is administered as when the arsenious acid has been swallowed alone." The case, recorded by Vandendale, in illustration of the efficacy of sulphuret of potash, is not detailed with sufficient correctness to invalidate this evidence. The reports of *sulphuretted hydrogen* are more favorable. Numerous experiments prove that the new compound, formed in the stomach by sulphuretted hydrogen and *liquid* arsenious arsenic, may be taken with impunity in somewhat large doses. Now if we remark that hydro-sulphuretted water may be largely swallowed without inconvenience; and that it acts *promptly* on *liquid* arsenious acid at a temperature lower than that of the human body; it must be concluded that this is the antidote of arsenious acid in a *liquid* state. But it is not so when the *solid* poison has been swallowed. The experiments, instituted by Renault, shew that in such cases, it is useless: and it unfortunately happens that arsenic, when employed with the intention of destroying life, is almost always taken in a solid form. The same objection applies to *lime-water;* which forms with *liquid* arsenious acid an insoluble and almost inert compound (arsenite of lime) but combines too slowly with the solid poison to be of service. *Acetic acid* can only dissolve the arsenious at the point of ebullition; and acetate of arsenic possesses properties as destructive as the oxyde.

To procure the expulsion of the poison by vomiting should, therefore, constitute the object of the physician. Copious ingestion of warm water, milk, sugared water, and decoctions of mucilaginous plants, may be prescribed with this view. Irritation of the throat with a feather or finger ought never to be neglected. Accumulated evidence is adduced to prove, that the destructive operation of the poison is less vehement when it has been taken into a full stomach; and signally mitigated by the liberal administration of diluent drinks and injections. When the patient cannot vomit, the stomach should be emptied by the elastic gum apparatus. Strong emetics serve only to aggravate the irritative effects of the poison. All fat and oily substances are pernicious: for animals are more speedily destroyed by arsenic mixed with butter or fat, than by the poison swallowed alone, or otherwise combined. *Theriaca* possesses no just claim to the character of an antidote: and the various vegetable infusions, proposed by Chansarel, are indebted solely for their virtues to the aqueous vehicle, and by no means preferable to simple warm water.

Abstraction of blood by lancet and leeches; immersion of the body, total or partial, in the warm bath; emollient fomentations and injections; antispasmodics and narcotis, are the remedies to be employed when abdominal inflammation has been developed, or the patient is assailed by alarming nervous symptoms. But success will mainly depend on the regimen observed by the patient during his protracted convalescence. He should subsist principally on milk, gruel, cream, rice, and mild liquids.

Here our analysis would have terminated for the present; but we cannot quit the subject without adverting to the fate of the unfortunate Gehlen,* who, a few months since, perished from inconsiderately exposing himself to the action of *arseniated hydrogen gas.* "Scarcely," says Ruhland, the friend and colleague of the deceased philosopher, in his letter to Guyton-Morveau, "had an hour elapsed" (from the exposure) "when he was attacked with incessant vomiting, shiverings, and alarming weakness. He died in my arms, after nine days of dreadful suffering—a martyr to his zeal for the progress of science." The remedies, principally employed, were milk and alkalies.

In our ensuing Number, we shall conclude the analysis of M. Orfila's first volume.

### PART III.

### SELECTIONS.

### FOREIGN JOURNALS.

#### *Memoir on a Case of poisoning by Opium.*†

* Adolph Ferdinand Gehlen, Fellow of the Academy of Sciences of Munich, and editor of a periodical work, entitled, *Journal fur die Chemie und Physik.* See *Annales de Chimie.* Tome xcv. Page 110. Rev.

